# Clustering of classical swine fever virus isolates by codon pair bias

**DOI:** 10.1186/1756-0500-4-521

**Published:** 2011-11-29

**Authors:** Immanuel Leifer, Dirk Hoeper, Sandra Blome, Martin Beer, Nicolas Ruggli

**Affiliations:** 1 Institute of Virology and Immunoprophylaxis (IVI), Sensemattstrasse 293, CH-3147 Mittelhäusern, Switzerland; 2 Institute of Diagnostic Virology, Friedrich-Loeffler-Institut, Südufer 10, 17493 Greifswald-Insel Riems, Germany

## Abstract

**Background:**

The genetic code consists of non-random usage of synonymous codons for the same amino acids, termed codon bias or codon usage. Codon juxtaposition is also non-random, referred to as codon context bias or codon pair bias. The codon and codon pair bias vary among different organisms, as well as with viruses. Reasons for these differences are not completely understood. For classical swine fever virus (CSFV), it was suggested that the synonymous codon usage does not significantly influence virulence, but the relationship between variations in codon pair usage and CSFV virulence is unknown. Virulence can be related to the fitness of a virus: Differences in codon pair usage influence genome translation efficiency, which may in turn relate to the fitness of a virus. Accordingly, the potential of the codon pair bias for clustering CSFV isolates into classes of different virulence was investigated.

**Results:**

The complete genomic sequences encoding the viral polyprotein of 52 different CSFV isolates were analyzed. This included 49 sequences from the GenBank database (NCBI) and three newly sequenced genomes. The codon usage did not differ among isolates of different virulence or genotype. In contrast, a clustering of isolates based on their codon pair bias was observed, clearly discriminating highly virulent isolates and vaccine strains on one side from moderately virulent strains on the other side. However, phylogenetic trees based on the codon pair bias and on the primary nucleotide sequence resulted in a very similar genotype distribution.

**Conclusion:**

Clustering of CSFV genomes based on their codon pair bias correlate with the genotype rather than with the virulence of the isolates.

## Background

Classical swine fever (CSF) is a serious and highly contagious disease of pigs that can cause important economical losses in the pig industries [1,2]. The disease is caused by the classical swine fever virus (CSFV), currently endemic in wild boars and in part also in domestic pigs in Asia, South America, and parts of Central and Eastern Europe [1,3,4]. Depending on the isolate, the disease can vary from an acute hemorrhagic fever to a chronic or unapparent infection. An acute infection with a highly virulent strain manifests with high fever, respiratory and gastrointestinal symptoms, multiple haemorrhages, neurological disorders, and a high mortality rate [5]. Chronic infections may not be immediately recognized due to the mild symptoms. Infections with low virulent isolates can remain unapparent. Thus CSFV viruses are divided into strains of highly, moderately, and low to avirulent strains (mainly vaccine strains) [6,7], see also Table 1 with the references therein. A number of live attenuated vaccines are available. These vaccines are mostly based on the Chinese vaccine strain (C-strain) and are completely avirulent [8-10].

**Table 1:** Overview of the CSFV strains used for this study.

Isolate	Genotype	Virulence status	GenBank (NCBI) entry	Reference
ALD D49532 hv	1.1	hv	D49532	[11]

Alfort187 × 87939 hv	1.1	hv	X87939	[12]

AlfortA19 U90951 hv	1.1	hv	U90951	[13]

Brescia AF091661 hv	1.2	hv	AF091661	[14]

Brescia M31768 lv^1^	1.2	hv	M31768	[14]

BRESCIAX AY578687 hv	1.2	hv	AY578687	[13]

CAP X96550 lv^2^	1.1	Low virulent	X96550	[15]

cF114 AF333000 hv	1.1	hv	AF333000	[16]

Eystrup AF326963 hv	1.1	hv	AF326963	[17]

Eystrup NC_002657 hv	1.1	hv	NC_002657	[17]

Glentorf U45478 lv^3^	1.1	Low virulent	U45478	[18]

JL106 EU497410 hv	1.1	hv	EU497410	[19]

Koslov HM237795 hv	1.1	hv	HM237795	[19]

Shimen AF092448 hv	1.1	hv	AF092448	unpublished

Shimen-HVRI AY775178 hv	1.1	hv	AY775178	[19]

SWH DQ127910 hv	1.1	hv	DQ127910	[6]

C_strain AY259122 va	1.1	va	AY259122	[17]

C_strain AY382481 va	1.1	va	AY382481	unpublished

C_strain AY663656 va	1.1	va	AY663656	unpublished

C_strain C-ZJ-2008 va	1.1	va	HM175885	unpublished

C_strain HCLV AF531433 va	1.1	va	AF531433	unpublished

C_strain HVRI AY805221 va	1.1	va	AY805221	unpublished

C_strain U45477 va	1.1	va	U45477	unpublished

C_strain Z46258 va	1.1	va	Z46258	[20]

flc-LOM EU915211 va	1.1	va	EU915211	

GPE- D49533 va	1.1	va	D49533	[11]

HCLV AF091507 va	1.1	va	AF091507	[21]

India vaccine EU857642 va	1.1	va	EU857642	unpublished

LOM EU789580 va	1.1	va	EU789580	[22]

LPC AF352565 va	1.1	va	AF352565	[23]

RUCSFPLUM AY578688 va	1.2	va	AY578688	[13]

Thiverval EU490425 va	1.1	va	EU490425	[24]

944IL94TWN AY646427 mv	3.4	mv	AY646427	[25]

Alfort-Tuebingen J04358 mv	2.3	mv	J04358	[26]

Borken GU233731 mv	2.3	mv	GU233731	[3]

CSF 39 AF407339 mv^4^	recombinant	mv-hv	AF407339	[27]

Euskirchen GU233732 mv	2.3	mv	GU233732	[3]

GXW_Z02 AY367767 mv	2.1	mv	AY367767	[27]

Hennef GU233733 mv	2.3	mv	GU233733	[3]

Jambul CSF0864 mv	2.3	mv	HQ148062	[28]

Novska CSF0821 mv	2.3	mv	HQ148061	[28]

Paderborn GQ902941 mv	2.1	mv	GQ902941	[29,30]

Penevezys CSF1048 mv	2.1	mv	HQ148063	[28]

Roesrath GU233734mv	2.3	mv	GU233734	[3]

Sp01 FJ265020 mv	2.3	mv	FJ265020	unpublished

Uelzen GU324242 mv	2.3	mv	GU324242	[3]

96TD AY554397 uk	2.1	uk	AY554397	unpublished

0406CH01TWN AY568569 uk	2.1	uk	AY568569	unpublished

HEBZ GU592790 uk	2.1	uk	GU592790	unpublished

SXCDK GQ923951 uk	2.1	uk	GQ923951	unpublished

SXYL2006 GQ122383 uk	2.1	uk	GQ122383	unpublished

ZJ0801 FJ529205 uk	2.1	uk	FJ529205	unpublished

Virulence status: highly virulent (hv), moderately virulent (mv), low virulent (lv) or vaccine strains (va), and unknown virulence (uk) is indicated according to the information available. If available, the references to the sequences are indicated.

^1 ^Brescia M31768 lv^1 ^is representing the sequence of strain Brescia C1.1.1 which is a low virulent strain obtained after the 30^th ^passage of strain Brescia on PK-15 cells [31].

^2 ^CAP X96550 lv^2 ^is described as highly virulent strain in some publications, but was originally described as cell culture adapted strain of low virulence [15].

^3 ^Glentorf U45478 lv^3 ^is described as low virulent or as highly virulent strain, depending on the report. In this study it is considered to be low virulent according to the publication of Handel et al. and Ahrens et al. [18,32]

^4 ^CSF 39 AF407339 mv^4 ^is a recombinant CSFV from China [27]. The virulence of this strain cannot be related to a particular genotype because the 5'NTR and the 3'NTR as well as the NS5A/B genes are homologous to genotype 1.1 strains, while the structural genes are homologous to genotype 2.1 strains. Furthermore the sequence of the original isolate is not known since the 32^nd ^cell culture passage was used for sequence analysis.

CSFV is classified within the genus *Pestivirus *of the family *Flaviviridae *together with Border disease virus (BDV) and Bovine viral diarrhoea virus (BVDV) [33]. Pestiviruses possess a single-stranded positive-sense RNA genome of approximately 12300 nucleotides, with 5'-terminal and 3'-terminal non-translated regions (5'-NTR, 3'-NTR) [34]. The genome encodes one polyprotein that is co- and post-translationally processed by the viral proteases N^pro^, NS2, NS3, and by cellular proteases [34]. The polyprotein is cleaved in the four structural proteins C, E^rns^, E1, E2, and in the eight non-structural proteins N^pro^, p7, NS2, NS3, NS4A, NS4B, NS5A, and NS5B [35-37].

By phylogenetic classification, the CSFV strains are divided into the three different genotypes 1, 2, and 3, every genotype consisting of three to four subgenotypes [38]. The most recent CSFV outbreaks in the European Community are associated essentially with isolates that cluster in the genotype 2. In general, the genotype 2 isolates are of moderate or low virulence, as are the isolates of genotype 3 [3,4,39]. To the best of our knowledge, the CSFV strains with the highest virulence identified so far all belong to genotype 1. However, there is no absolute relationship between genotype and virulence, as there are also low virulent field isolates (e.g. "Glentorf") of genotype 1, and isolates within the genotype 2 group (e.g. "Uelzen") for which infection of piglets results in higher mortality than with most genotype 2 isolates. In addition, all vaccine strains belong also to genotype 1, as they were derived from genotype 1 strains by attenuation through multiple passages in non natural hosts, typically rabbits and guinea pigs, or in cell cultures derived from them.

Various experimental approaches were implemented with the aim of identifying the virulence determinants related to a particular CSFV isolate. Numerous mutants with deletions, insertions, peptide or amino acid exchanges were analyzed and described in detail [40-46]. All mutants described so far were attenuated, leading to the conclusions that the modified positions may be relevant for the virulence of a specific strain or of CSFV in general. Certainly, strain-specific virulence factors determine whether an infection results in acute hemorrhagic fever, chronic disease or subclinical infection. Whether virulence determinants can be associated with particular amino acid positions remains unanswered. From a general point of view however, one may speculate that virulence depends mostly on the speed and level of virus replication. For poliovirus and influenza virus it was shown that the codon pair bias can influence fitness and virulence [47,48]. The codon pair bias refers to the non random juxtaposition of codons, while the non random usage of synonymous codons for the same amino acids is referred to as codon bias. Previous studies showed that differences in synonymous codon usage did not relate to the virulence of CSFV isolates [7]. There is no analysis of codon pair usage of CSFV available. Therefore, the aim of this study was to investigate whether the codon pair usage of CSFV may relate to virulence or simply cluster the isolates into their genotype.

## Results

### Sequencing of complete genomes of recent CSFV isolates

**Figure 1:**
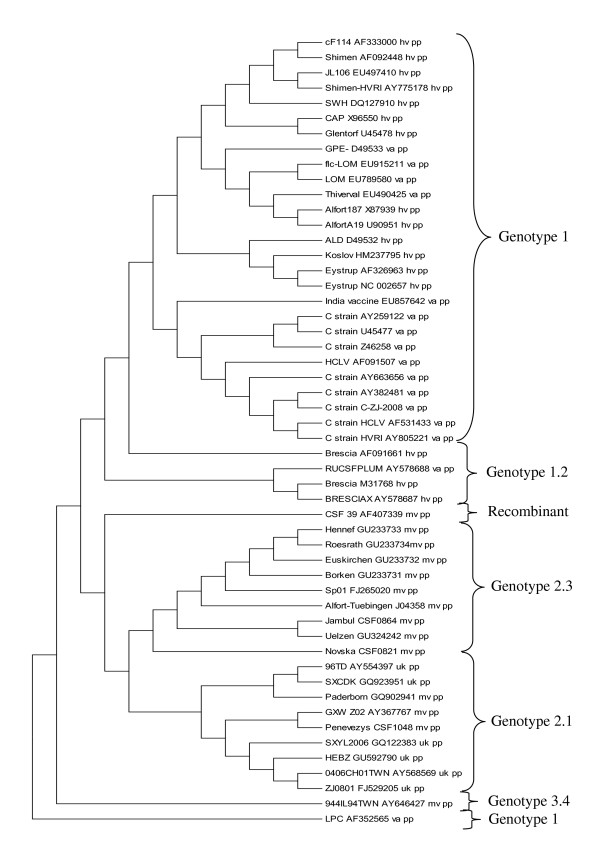
**Phylogenetic tree representing 52 complete CSFV polyprotein encoding nucleotide sequences**. The tree was built using the MEGA4 software.

In order to include some of the latest European CSFV isolates in the codon pair bias analysis, the genome of three recent field isolates were sequenced. The complete nucleotide sequences of the isolates CSFV/2.3/dp/CSF0821/2002/HR/Novska, CSFV/2.3/dp/CSF864/2007/BG/Jambul, and CSFV/2.1/dp/CSF1048/2009/LT/Penevezys were deposited to the NCBI GenBank nucleotide database [GenBank: HQ148061-HQ148063]. The genomes of the newly sequenced isolates encode a polyprotein of 3898 amino acids. The 5'NTRs of the three isolates are 373 nucleotides long. The 3'NTR is composed of 225 nucleotides for the "Novska" isolate and of 226 nucleotides for the two other isolates. These three sequences were included in a phylogenetic tree together with 49 complete CSFV genome sequences obtained from GenBank (Figure 1). The three newly sequenced isolates belong to the genotype 2, with the isolates "Novska" and "Jambul" clustering with the subgenotype 2.3 strains and the isolate "Penevezys" belonging to subgenotype 2.1. With experimental infections of pigs, the isolate "Penevezys" was classified as low to moderately virulent whereas the two other isolates were moderately virulent. Detailed information on the genotype, virulence, and origin of the 52 CSFV isolates analysed are provided in Table 1.

### The relative synonymous codon usage (RSCU) does not vary among different CSFV isolates

**Figure 2:**
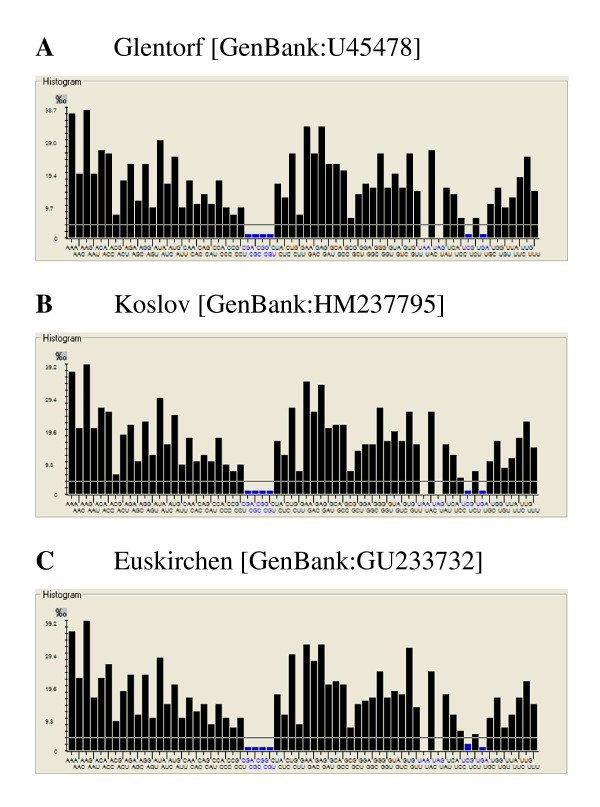
**Relative synonymous codon usage exemplified with three prototype CSFV isolates**. The histograms show the frequencies of synonymous codon usage in per mille for the strains "Glentorf" [GenBank:U45478], "Koslov" [GenBank:HM237795], and "Euskirchen" [GenBank:GU233732]. The values were calculated using the ANACONDA 2.0 software.

In order to determine the variations in RSCU between CSFV isolates of different genotypes and virulence, the frequency of each codon was determined for the 52 complete genome sequences available. As an example, the codon usage of three prototype isolates of different virulence, the low virulent "Glentorf" strain, the highly virulent "Koslov" strain, and the moderately virulent "Euskirchen" isolate is shown in Figure 2A-C . All three virus isolates have a very similar RSCU pattern. The two codons encoding the amino acid lysine (AAA and AAG) are the most frequent codons appearing in the CSFV genomes. The AAG triplet is slightly preferred. AAA is found in average 142,4 times/polyprotein with a standard deviation of 3.8 whereas AAG is found in average 151,6 times/polyprotein with a standard deviation of 3.6, independently of genotype and virulence. For the amino acid arginine there is a total of six different codons possible: CGA, CGC, CGG, CGU, AGA, and AGG. The four codons CGA, CGC, CGG, CGU are amongst the rarest codons used in all isolates. Thus, arginine is encoded almost exclusively by AGA and AGG, but here again, no major differences between strains of different virulence can be observed. Overall, no significant differences were observed between the different isolates confirming earlier results showing that the RSCU does not vary between strains of different virulence [7].

### The codon pair bias clusters CSFV into groups of different genotypes

**Figure 3:**
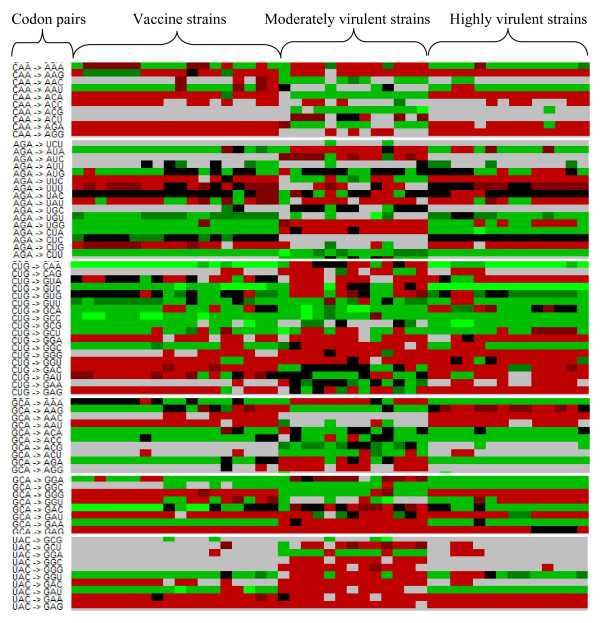
**The codon pair usage clusters CSFV strains into their different genotypes**. Parts of a codon pair context map alignment of 46 different CSFV isolates with known virulence status are shown. The virus isolates are divided into vaccine strains, moderately virulent strains, and highly virulent strains. The frequencies of codon pair usage are represented by different colours. Green indicates that codon pairs are strongly preferred. Red indicates that codon pairs are rejected. Black means that there is no significant difference in codon pair usage. The values were calculated using the ANACONDA 2.0 software.

**Figure 4:**
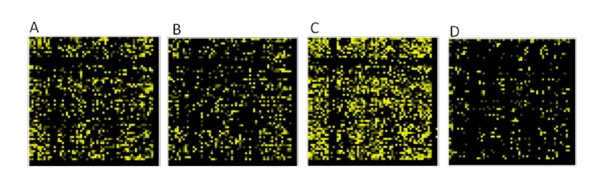
**Codon pair bias overlays of vaccine and highly virulent CSFV strains using the differential display codon pair context tool of ANACONDA 2.0; 61 × 64 codon pair bias matrices are shown**. Yellow spots indicate differences in the corresponding codon pair usage whereas black colour means that codon pairs are used with similar residual values. Shown are codon pair overlays of the "GPE^-^" vaccine strain and the parental highly virulent "ALD" strain (**A**), of the highly virulent "ALD" and "Koslov" strains (**B**), of the "GPE^-^" and "C-strain Riems" vaccine viruses (**C**), and of the "C-strain Riems" and "HCLV" vaccine viruses (**D**).

Since the analysis of RSCU did not reveal any obvious differences among isolates of different virulence, it was of interest to determine whether the codon pair usage differs between CSFV isolates. To this end, the ANACONDA 2.0 software was applied to analyse the codon pair bias of the polyprotein encoding sequences of the 52 CSFV isolates. As opposed to the RSCU, clear differences were observed between different isolates (Figure 3). The codon pair analysis clustered the isolates in two groups, one representing the avirulent and the highly virulent strains, and the other the moderately virulent strains. The codon pairs CAA-AGA and GCA-GGG for instance are preferred by moderately virulent strains, but strongly rejected among vaccine viruses and highly virulent strains. On the other hand, the pairs UAC-GGU, UAC-GAU, AGA-CUA, and GCA-GAA are preferred by vaccine and highly virulent viruses and are rejected by moderately virulent strains. Remarkably, the codon pairs with the pattern UAC-GGN and UAC-GAN strongly rejected among moderately virulent strains. Pairwise comparison of the complete codon pair context maps of vaccine strains among each other, and those of vaccine and highly virulent strains revealed similar degrees of variability (exemplified in Figure 4A-D). Interestingly, the diversity within the group of vaccine strains is in some cases higher compared to the diversity between vaccine strains and highly virulent strains (Figure 4A-D).

It was also hypothesised that the codon pair bias may affect specifically the genome replication efficiency. In order to determine whether the codon pair bias differs between the replicase and the structural proteins, which would suggest a potential effect of the codon pair bias on replication efficiency and virulence, artificial open reading frames ORFs were constructed covering the structural proteins and the NS5B protein of each CSFV strain. These ORFs were compared with respect to codon pair usage. No obvious differences in codon pair usage between structural and replicase genes were found, irrespectively of genotype and virulence (data not shown). Therefore, analysis of the individual genes did not allow discrimination between virulence either.

**Figure 5:**
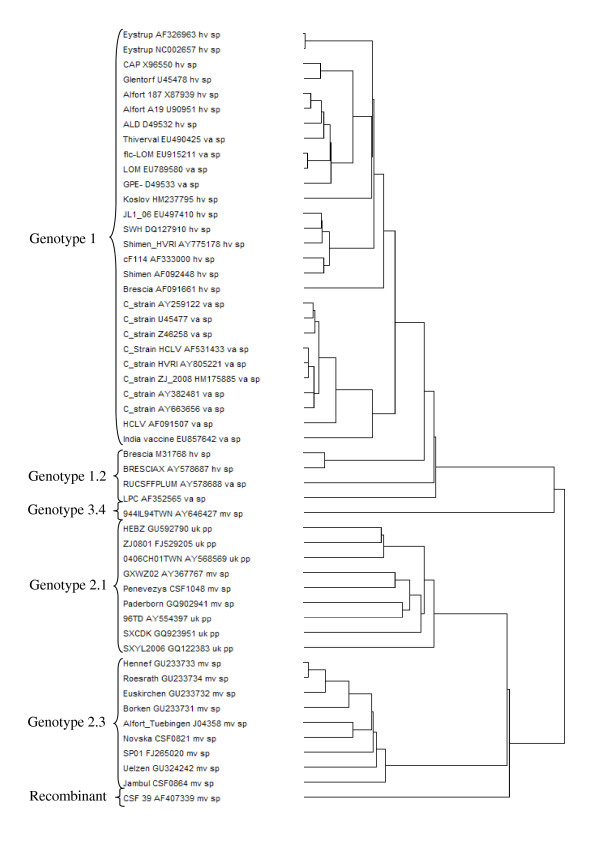
**Phylogenetic tree of the CSFV polyprotein sequence alignment of 52 different isolates based on their codon pair bias, for analysis the ANACONDA 2.0 software was used**.

Finally, a phylogenetic tree based on the codon pair usage of the complete polyprotein encoding nucleotide sequences of the 52 isolates was constructed using the ANACONDA 2.0 software (Figure 5). The codon pair usage clusters the isolates in genotypes 1, 2, 3, and subgenotypes 2.1 and 2.3, similarly to the phylogenetic tree based on the primary nucleotide sequence (compare Figures 1 and 5). Interestingly, some vaccine strains are grouped with the highly virulent strains, e. g. the strains "Alfort" and "Thiverval". According to these data, the codon pair bias clusters the CSFV isolates by genotype rather than by virulence.

## Discussion

Despite numerous efforts, CSFV virulence could not be linked to any particular genome sequence signature so far. Most if not all highly virulent CSFV strains belong to genotype 1 as do the vaccine strains (Table 1 and the references listed therein). Moderately virulent strains belong essentially to genotypes 2 and 3. The genetic variability within the genotype 1 is lower compared to strains of genotype 2 and 3 [49,50]. This lets hypothesize that sequence signatures of virulence may be found, especially with full sequence data of vaccine strains and parental highly virulent strains [51].

From the functional point of view, virulence may depend on viral replication efficiency, which can be influenced by differences in protein expression. Codon and codon pair bias can have an impact on translation efficiency and protein expression as it was shown for bacteria and yeast [52,53]. For poliovirus and influenza A virus, the artificial use of rare codons and of underrepresented codon pairs reduced viral protein translation and viral fitness, resulting in virus attenuation in vivo [47,48]. Consequently, a potential influence of codon usage and in particular of codon pair usage on CSFV virulence was considered. The analysis of the RSCU of 52 virus isolates covering the whole spectrum of virulence did not reveal any relationship with virulence. This confirmed earlier results obtained with the complete genome sequences of 35 isolates [7]. Thus codon usage between CSFV isolates is very similar, which is in agreement with the findings that RNA viruses of the same host category have the same codon usage preferences [54]. For the human immunodeficiency virus type-1, the RSCU is different from that of the human host. Adaptation towards human RSCU was attributed to the homogenization of the codon usage by mutation pressure rather than host adaptation [55].

Analysis of the codon pair bias of the complete coding sequence of the 52 isolates revealed a clear clustering (Figure 3). Vaccine strains and highly virulent strains showed mostly the same pattern, differing from the codon pair usage of moderately virulent strains. Because highly virulent and vaccine strains belong to genotype 1 and moderately virulent strains belong essentially to genotype 2, similarities in codon pair usage within a genotype might be due to the high proportion of sequence identity. Indeed, the genotype clustering obtained with phylogenetic analysis based on the codon pair usage and on the primary nucleotide sequence was nearly identical (compare Figures 5 and 1). Nevertheless, this does not exclude a possible relationship of the codon pair usage with the virulence phenotype. The codon pair UAC-GNN for instance is less preferred by CSFV strains of genotype 2. Cytosin-phosphatidyl-Guanin (CpG) dinucleotides are signals for DNA methylation in eukaryotes and regulate gene expression [56,57]. A reduction of UAC-GNN codon pair usage could reflect a host-specific adaption, as it might influence the host anti-viral response as described for other viruses [58,59]. For CSFV it is unknown whether adaptation to the host is linked to a gain of viral fitness. One could hypothesize that highly virulent CSFV strains would emerge through increased viral replication in the host. However, adaptation to the host is likely to result in optimized rather than in enhanced replication since occurrence of higher virulent strains has not been observed in CSFV field isolates during the last years [3,4,39,60]. From the evolutionary point of view, natural selection or adaptation towards a moderately virulent strain makes sense, because the mortality of the host is lower [39,60,61]. In addition, failure in early diagnosis due to mild clinical symptoms contributes to the dissemination and survival of the virus [59]. Thus, a moderately or low virulent virus has a greater chance of circulating in a pig or wild boar population without being detected [62-64]. Hence the reduced virulence observed with the CSFV isolates from the more recent outbreaks in Europe could result from several driving forces representing advantages for the virus. In fact, during the last decades CSFV outbreaks in Europe and Asia were increasingly caused by genotype 2 and 3 isolates, while the older CSFV field isolates belong to genotype 1. This suggests that evolution of CSFV is directed towards genotype 2 and 3. However, it is unknown if this is applicable to South American isolates since sequence information is missing. The development of live attenuated CSFV vaccine strains was based on isolates belonging to the genotype 1, which explains the close phylogenetic relationship between highly virulent and vaccine strains within the same genotype. Interestingly, there are nevertheless obvious differences in codon pair usage among strains of genotype 1 as seen from the overlays of codon pair matrices. These differences are the most prominent between the two unrelated "GPE^-" ^and "C-strain" vaccine strains attenuated in guinea pigs and rabbits, respectively. It is likely that these differences are in part caused by the propagation of the viruses in different hosts.

## Conclusions

The present results describe the first extensive codon pair bias analysis of a representative number of CSFV isolates covering the complete spectrum of virulence. Overall, the CSFV strains can be grouped in two main clusters according to the codon pair usage. Thus codon pair bias analysis can support CSFV phylogeny. However, based on the data presented here, a direct link between the codon pair usage and CSFV virulence cannot be established.

## Methods

### Sequencing of complete CSFV genomes

Nucleotide sequence analysis of complete CSFV genome was performed by pyrosequencing with a FLX Genome Sequencer (Roche Diagnostics, Mannheim, Germany) as described previously [3]. Briefly, full CSFV genome DNA fragments (obtained by long-range RT-PCR) were separated by agarose gel electrophoresis and purified using the Zymoclean™ Gel DNA Recovery Kit (Zymo Research Corporation, Orange, CA, USA) prior to analysis with the FLX Genome Sequencer. The 5'NTR and 3'NTR were sequenced using commercial kits for RACE RT-PCR (5'RACE System and 3'RACE System, Invitrogen, Carlsbad, CA, USA) according to the manufacturers recommendations. Minor modifications were performed as described previously [3]. The raw sequence data were assembled using the GS assembler software newbler (v. 2.0.00.22; Roche, Mannheim). The nucleotide sequence information was deposited to the NCBI GenBank nucleotide database [65].

### Sequence data source and additional sequence information

Complete genome sequences of 49 different CSFV isolates were obtained from the NCBI GenBank nucleotide database. Detailed information on the virus isolates is provided in Table 1. Virus isolates were grouped in highly virulent (hv), moderately virulent (mv), and low virulent (lv) or vaccine strains (va). According to the information available, 46 virus isolates were subdivided into these three groups composed of 16 highly virulent, 14 moderately virulent, and 16 vaccine strains (Table 1). For the remaining virus isolates virulence could not be determined.

### Analysis of RSCU and codon pair usage

The relative synonymous codon usage is expressed as RSCU value of a codon [53]. The RSCU value expresses the relationship between the observed and the expected codon frequency and was calculated with the ANACONDA 2.0 software (Universidade de Aveiro, Portugal) [66]. The codon context bias of the complete polyprotein encoding nucleotide sequence of 52 different CSFV isolates was investigated using the software package ANACONDA 2.0 as described [67-69]. In addition, different regions of the genomes were analysed separately. To this end, artificial ORFs for the NS5B and the structural protein genes were constructed by adding a start and stop codon to the corresponding coding regions. Codon pair biases were analysed according to their relative occurrence. Statistical calculation of the codon pairs is given in relation to its real occurrence and the expected incidence independently of their distribution. The ANACONDA 2.0 software displays a codon pair context map for each viral ORF. This context map consists of 3904 possible codon pairs given in a vertical raw with one coloured square for each codon pair. The colours represent the frequency of occurrence: red coloured squares indicate codon pairs that are strongly rejected, whereas preferred codon pairs are represented in green colour. Codon pairs represented by black squares are statistically not significant.

Phylogenetic trees based on codon pair bias were created with the ANACONDA 2.0 software. Neighbour-joining trees with the maximum composite likelihood method using complete polyprotein encoding nucleotide sequences were constructed with the MEGA4 software (Molecular Evolutionary Genetics Analysis, Center for Evolutionary Medicine and Informatics, Tempe, USA) software [69].

## Conflict of interests

The authors declare that they have no competing interests.

## Authors' contributions

IL has done the statistical analyses and written the manuscript. NR is responsible for the design and supervision of the project and for the manuscript. DH has done most parts of the sequencing and MB has been involved in carefully revising the manuscript and giving substantial input during interpretation of the data. SB has determined the virulence phenotype of selected CSFV isolates in pigs. All authors have read and approved the final manuscript.
